# Network-based performance analysis of multidimensional performance characteristics in trained male youth combat athletes

**DOI:** 10.3389/fspor.2026.1932190

**Published:** 2026-08-31

**Authors:** Sadanan Kernluea, Phornpot Chainok, Piyathida Thongchai, Kanapan Pachatikapanya, Sirinapha Noiphon, Jian Zhi Lin, Paulo Felipe Ribeiro Bandeira, Rodrigo Zacca

**Affiliations:** 1 Faculty of Sport Science, Burapha University, Chonburi, Thailand; 2 Faculty of Sport Science and Human Performance, Institute of Entrepreneurial Science, Sathon, Thailand; 3 Department of Physical Education, National Taiwan University of Sport, Taichung, Taiwan; 4 Universidade Regional do Cariri, Crato, Brazil; 5 Research Center in Physical Activity, Faculty of Sports, Health and Leisure (CIAFEL), University of Porto (FADEUP), Porto, Portugal; 6 Laboratory for Integrative and Translational Research in Population Health (ITR), Porto, Portugal; 7 Nucleus of Research in Human Motricity Sciences, Universidad Adventista de Chile, Chillán, Chile; 8 Laboratory of Sport Physiology, Faculty of Sports, University of Porto, Porto, Portugal

**Keywords:** athlete development, biological maturation, combat sports, executive function, Gaussian graphical model, youth athletes

## Abstract

Youth combat sport performance relies on a complex interplay of biological maturation, anthropometrics, neuromuscular capacity, and executive cognitive function. Despite this, these domains are typically studied in isolation. To bridge this gap, the present study applied a network-based performance analysis to uncover the multidimensional organization of these characteristics in trained male youth combat athletes. We evaluated biological maturation, anthropometry, neuromuscular performance and executive cognitive function in eighty-eight trained male athletes (13–16 years) from six combat sport disciplines. A Gaussian graphical model was estimated with the Extended Bayesian Information Criterion graphical least absolute shrinkage and selection operator (EBICglasso; *γ* = 0.25). Nonparametric and case-dropping bootstrap procedures were used to evaluate network accuracy and stability. The estimated network contained 35 variables with 129 of 595 possible edges (network density = 21.7%). Anthropometric and maturational characteristics, particularly skeletal muscle mass, body mass, body height and maturity offset, were found to be the primary structural components, whereas Trail Making Test A completion time and Five-Key Task incongruent accuracy occupied highly connected positions within the network, linking cognitive, anthropometric and neuromuscular domains. The strongest conditional associations were observed between countermovement jump height from flight time and velocity (weight = 0.64; 95% CI: 0.57–0.77) and maturity offset and chronological age (weight = 0.59; 95% CI: 0.54–0.81). Strength centrality had acceptable stability (CS = 0.60). The findings suggest that performance in youth combat athletes is organized as an integrated multidimensional system of conditional associations. These results support the use of network-based performance analysis as a systems-oriented framework for multidimensional athlete profiling and hypothesis generation, while longitudinal studies are required to determine developmental trajectories and evaluate potential training applications.

## Introduction

Combat sports represent ancient traditions of self-defense and ritual that have influenced global societies for millennia (1). In the modern era, many different disciplines, such as boxing, wrestling and judo, have diversified into highly regulated combat sports. This transition has been characterized by the implementation of standardized scoring systems and safety regulations designed to preserve traditional foundations while enhancing athletic performance (2, 3). Achieving high performance standards requires an integrated interaction of physical strength, neuromuscular coordination and executive functions (4, 5). Therefore, achieving these complex athletic attributes necessitates the implementation of organized and age-appropriate long-term development paradigms (6).

From the perspective of youth combat sport performance, at the fundamental basis of these development paradigms is the improvement of cognitive abilities including those such as attention, reaction time and decision-making that enable elite athletes to analyze intricate situational data and predict opponents' actions under pressure (7, 8). Besides, integrating sport-specific exercises into neuromuscular conditioning in long-term training enhances agility and executive control, essential for tactical adaptation in dynamic environments (4, 9). Taken together, this systematic strategy provides that athletes maximize performance efficiency and respond effectively in the high-pressure context of modern competition (10, 11).

Despite their importance, most of the current research has concentrated on adult or elite athletes, resulting in little empirical evidence regarding the integrated neuromuscular and cognitive characteristics of youth populations (1, 12). During adolescence, the interplay between growth and maturation processes and sport-specific training stimuli reveals distinct developmental trajectories across young athletes highlighting the discipline-specific requirements (6, 13). The requirements of each combat sport correlate with the timing of peak height velocity (PHV), a crucial period in which accelerated development can affect neuromuscular function, coordination and susceptibility to injury (2, 13). Thus, as most youth-focused studies examine these performance categories in isolation, they neglect to consider the interplay of neuromuscular, anthropometric, maturational and cognitive factors as an integrated system during these crucial developmental periods.

To address these limitations, a methodological framework capable of capturing multidirectional and interdependent relationships is required. Performance network analysis provides a systems-oriented methodology by representing performance indicators as interrelated nodes inside a structured network of connections (14, 15). Such approaches facilitate the investigation of complicated and high-dimensional data without rigorous underlying assumptions, highlight conditional dependencies among variables, provide unambiguous visual representations of multivariate relationships and support generating theoretically informed hypotheses regarding the relationships among multidimensional performance indicators for subsequent longitudinal investigation (14, 16).

To address these limitations, a methodological framework capable of capturing multidirectional and interdependent relationships is required. Performance network analysis provides a systems-oriented approach by representing performance indicators as interconnected nodes and identifying conditional dependencies among anthropometric, maturational, neuromuscular and cognitive variables (15). This framework enables the exploration of complex multivariate relationships and supports hypothesis generation for subsequent longitudinal research. This study applied network analysis to characterize the multidimensional performance network of trained male youth combat athletes during adolescence by identifying conditional associations among performance domains and the most influential nodes within the network. It was hypothesized that neuromuscular, cognitive, anthropometric and maturational variables would form a highly interconnected performance network rather than independent clusters. Furthermore, executive cognitive function and neuromuscular performance variables were expected to exhibit the greatest network centrality, indicating their prominent role in coordinating the overall performance system in youth combat athletes.

## Materials and methods

### Participants

Eighty-eight trained male youth combat sport athletes (13–16 years) participated in this cross-sectional study. Participants were recruited from the national combat sports training center and represented six combat sport disciplines: judo (*n* = 18), jujitsu (*n* = 15), Muay Thai (*n* = 15), wrestling (*n* = 12), taekwondo (*n* = 15) and pencak silat (*n* = 13). The mean age of the participants was 14.58 ± 1.16 years (range: 13–16 years). The mean maturity offset was 0.81 ± 0.99 years, indicating that, on average, participants were estimated to be approximately 0.8 years after peak height velocity. Mean body height and body mass were 167.02 ± 9.38 cm and 56.29 ± 13.51 kg, respectively.

All athletes were actively engaged in structured training under the supervision of certified coaches and regularly participated in regional and national competitions. Participants had a minimum of two years of formal combat sport training and completed approximately 6–8 training sessions per week, with each session lasting approximately 60–90 min. All participants were free from injury or medical conditions that would have limited maximal physical performance at the time of testing. To minimize potential seasonal influences on performance, all assessments were conducted during the preparatory phase of the annual training cycle. Prior to enrolment, written informed consent was obtained from parents or legal guardians and written assent was obtained from all participating athletes in accordance with the approved ethics protocol. Detailed descriptive characteristics of the study sample are presented in Table 1.

**Table 1:** Descriptive statistics (mean ± SD and 95% confidence intervals) for the maturational, anthropometric, neuromuscular and cognitive variables included in the performance network analysis.

Variables	Domain	Mean ± SD	95% CI
Chronological age (yrs)	Maturity	14.58 ± 1.16	14.33–14.83
Maturity offset (yrs)	Maturity	0.81 ± 0.99	0.60–1.02
Right-hand 2D:4D	Maturity	0.99 ± 0.06	0.97–1.00
Left-hand 2D:4D	Maturity	0.98 ± 0.06	0.97–0.99
Difference (RF–LF)	Maturity	0.003 ± 0.03	−0.07–0.07
Body height (cm)	Anthropometric	167.02 ± 9.38	165.04–169.01
Body mass (kg)	Anthropometric	56.29 ± 13.51	53.43–59.15
Arm span (cm)	Anthropometric	165.96 ± 10.69	163.69–168.23
Body-mass index (kg·m⁻²)	Anthropometric	19.47 ± 3.25	18.79–20.16
Percent body fat (%)	Anthropometric	11.23 ± 5.76	10.00–12.45
Fat mass (kg)	Anthropometric	6.65 ± 4.67	5.66–7.64
Skeletal muscle mass (kg)	Anthropometric	27.66 ± 5.05	26.59–28.73
SRT_Avg	Cognitive Function	284.40 ± 83.96	266.61–302.19
SRT_Acc	Cognitive Function	92.10 ± 15.35	88.85–95.35
CRT_Avg	Cognitive Function	414.84 ± 56.39	402.89–426.79
CRT_Acc	Cognitive Function	79.25 ± 22.71	74.44–84.06
TMTA_Ct	Cognitive Function	37.31 ± 9.33	35.33–39.29
TMTA_Er	Cognitive Function	0.18 ± 0.65	0.04–0.32
TMTB_Ct	Cognitive Function	82.36 ± 33.89	75.18–89.54
TMTB_Er	Cognitive Function	9.02 ± 12.27	6.42–11.62
TMTB_B–A diff	Cognitive Function	43.91 ± 28.15	37.95–49.87
TMTB_B/A ratio	Cognitive Function	2.19 ± 0.69	2.04–2.33
FKTC_Avg	Cognitive Function	406.70 ± 67.80	392.34–421.07
FKTC_Acc	Cognitive Function	87.69 ± 18.04	83.87–91.52
FKTI_Avg	Cognitive Function	435.88 ± 106.14	413.39–458.36
FKTI_Acc	Cognitive Function	62.14 ± 28.67	56.06–68.21
DFT_Fd	Cognitive Function	7.34 ± 3.15	6.67–8.01
DFT_Ed	Cognitive Function	7.07 ± 3.78	6.27–7.87
DFT_Sd	Cognitive Function	2.77 ± 2.97	2.14–3.40
DFT_Tc	Cognitive Function	17.18 ± 7.79	15.53–18.83
MRT	Cognitive Function	6.73 ± 4.00	5.88–7.57
SVT	Cognitive Function	6.58 ± 3.94	5.75–7.41
Grip strength_D_PF (N)	Neuromuscular Performance	285.02 ± 79.90	268.09–301.95
Grip strength ND_PF (N)	Neuromuscular Performance	256.20 ± 72.73	240.79–271.61
Total grip strength_PF (N)	Neuromuscular Performance	541.22 ± 151.30	509.16–573.28
Grip_asymmetry (%)	Neuromuscular Performance	9.77 ± 7.05	8.28–11.26
Height jump FT (cm)	Neuromuscular Performance	26.23 ± 7.14	24.72–27.74
Height jump V (cm)	Neuromuscular Performance	23.99 ± 6.57	22.59–25.38
CMJ_D_PF (N)	Neuromuscular Performance	754.05 ± 203.01	711.03–797.06
CMJ_ND_PF (N)	Neuromuscular Performance	709.61 ± 190.96	669.15–750.07
CMJ_Total_PF (N)	Neuromuscular Performance	1,462.52 ± 394.99	1,378.83–1,546.21
CMJ_Asymmetry (%)	Neuromuscular Performance	5.77 ± 4.31	4.86–6.68
IMTP_D_PF (N)	Neuromuscular Performance	1,030.27 ± 299.71	966.77–1,093.77
IMTP_ND_PF (N)	Neuromuscular Performance	948.74 ± 281.78	889.03–1,008.44
IMTP_Total_PF (N)	Neuromuscular Performance	1,979.35 ± 576.96	1,857.10–2,101.60
IMTP_Asymmetry (%)	Neuromuscular Performance	7.74 ± 5.75	6.53–8.96
Lower-limbs_DSI	Neuromuscular Performance	0.77 ± 0.19	0.73–0.81
NHE_D_PF (N)	Neuromuscular Performance	236.56 ± 70.18	221.69–251.43
NHE_ND_PF (N)	Neuromuscular Performance	207.33 ± 62.32	194.12–220.53
NHE_Total_PF (N)	Neuromuscular Performance	443.89 ± 130.27	416.29–471.49
NHE_Asymmetry (%)	Neuromuscular Performance	11.90 ± 8.91	10.01–13.79

Data are mean ± SD [95% CI]. 2D:4D, second-to-fourth digit ratio; RF–LF, difference between right and left 2D:4D; SRT, simple reaction time; CRT, choice reaction time; TMT, Trail Making Test; TMT-B-A diff, time on TMT-B minus time on TMT-A; TMT-B/A ratio, time on TMT-B divided by time on TMT-A; FKT, Flanker Task (C, congruent; I, incongruent); DFT, Design Fluency Test (Fd, filled dots; Ed, empty dots; Er, error; Sd, switching dots; Tc, total correct designs); MRT, mental rotation test; SVT, spatial visualization test; Avg, average; Acc, accuracy (% correct responses); D, dominant limb; ND, non-dominant limb; PF, peak force; CMJ, countermovement jump; IMTP, isometric mid-thigh pull; DSI, dynamic strength index; NHE, Nordic hamstring exercise.

#### Procedures

**Figure 1:**
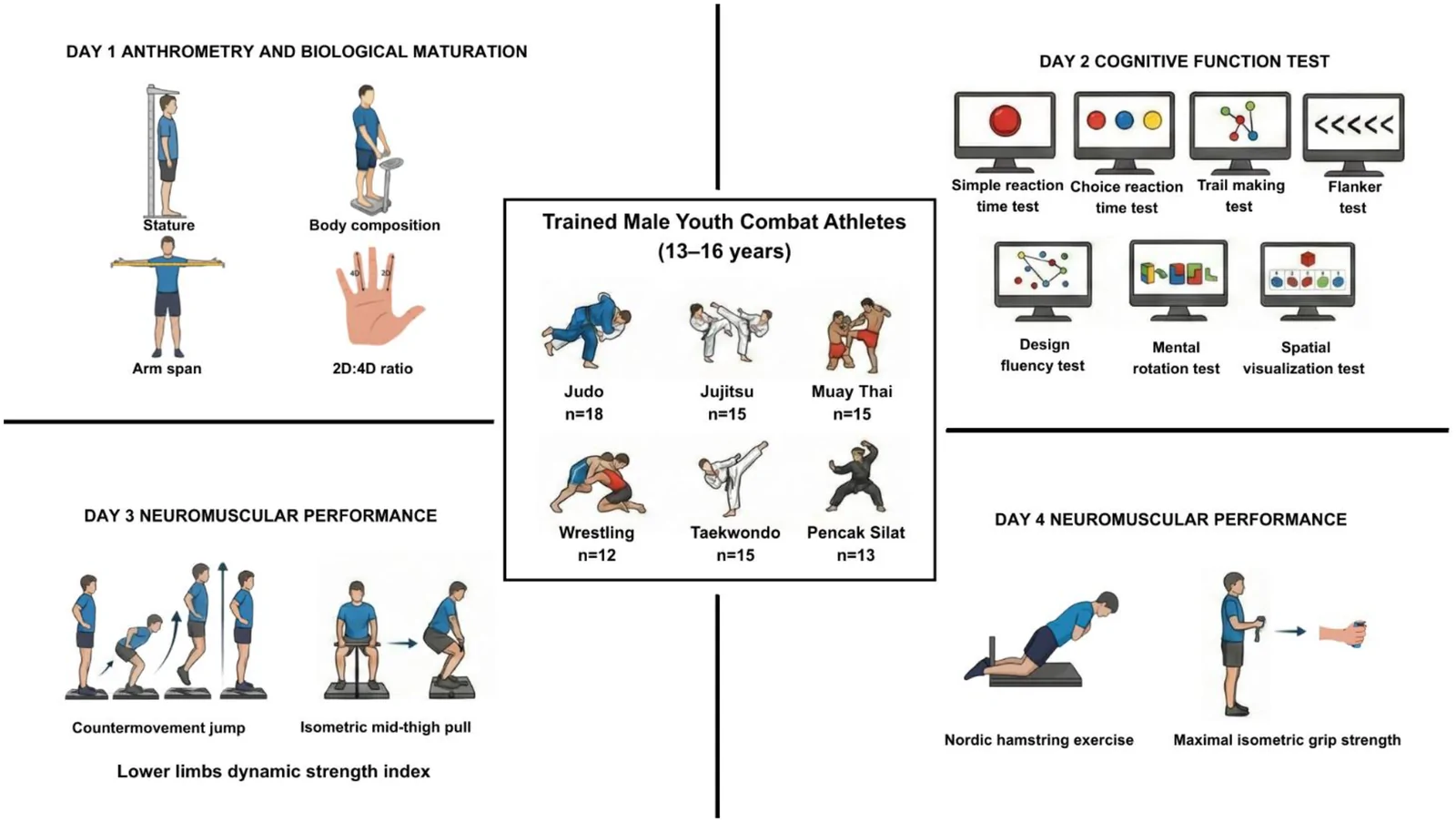
Conceptual overview of the study design illustrating the multidimensional assessment of anthropometric, maturational, cognitive and neuromuscular characteristics in trained male youth combat athletes.

A cross-sectional, multivariate profiling design was employed to systematically examine young trained combat athletes using a network-based performance analysis framework. The measurement was carried out through a structured four-day testing schedule with the purpose of assessing anthropometric characteristics, biological maturation, cognitive function and neuromuscular performance (Figure 1). The testing order was fixed for all participants to ensure standardized administration and reduce procedural variability. Besides, the four testing sessions were separated by 24 h intervals. Before data collection, all participants completed a familiarization session, receiving standardized instructions and practicing each cognitive and neuromuscular assessment. Participants were instructed to avoid strenuous exercise for 24 h before each testing session, refrain from caffeine-containing beverages for at least 12 h, consume their habitual diet, maintain normal hydration, obtain adequate sleep on the preceding night and attend testing at approximately the same time of day. Coaches were requested not to schedule high-intensity training immediately before testing sessions.

### Anthropometry, body composition, biological maturation and digit ratio

The first day was scheduled for baseline physical profiling. Anthropometric measurements were performed by an ISAK Level I anthropometrist following the standardized protocols of the International Society for the Advancement of Kinanthropometry (ISAK), then assessing biological maturation to determine the participants’ developmental status. A multi-frequency bioelectrical impedance analyzer (X­Contact 357S, Jawon Medical Co., Ltd., South Korea) was employed to evaluate anthropometric measurements and body composition. The apparatus included an ultrasonic stadiometer for height measurement and bioelectrical impedance analysis was employed to determine body mass, BMI and body fat percentage (17).

Biological maturation was estimated using the sex-specific maturity-offset equation developed by Moore et al. (18) for male adolescents (18). The maturity-offset value represents the estimated number of years before or after peak height velocity (PHV), whereas age at PHV was calculated by subtracting maturity offset from chronological age. Chronological age was determined by subtracting the participant's date of birth from the assessment date and dividing the result by 365.25 (19). Because maturity offset provides an indirect estimate of biological maturation relative to PHV rather than a direct assessment of maturity offset, the resulting values were interpreted as indicators of maturation timing rather than maturity offset stage.

The second-to-fourth digit ratio (2D:4D) was measured separately because it has been proposed as an indirect anthropometric marker of prenatal androgen exposure rather than a measure of current biological maturation. Digit lengths were measured bilaterally from the basal crease to the fingertip using a digital Vernier caliper (0.01-mm precision). Three independent measurements were obtained for each digit and the mean value was used to calculate the 2D:4D ratio for each hand.

### Cognitive function

On the second day, participants completed a systematic computerized cognitive function evaluation in a private, ecologically regulated environment, adhering to standardized verbal and visual instructions. Computerized cognitive testing was administered using the Department of Physical Education (DPE), Thailand, Computerized Cognitive Test Battery, which assesses cognitive functions relevant to sports performance. A seven-task battery evaluated executive function, processing speed and visuospatial abilities—domains recognized for distinguishing performance among six combat sports disciplines. The protocol encompassed simple and choice reaction time tasks for sensorimotor processing and rapid decision-making; Trail Making Tests (TMT-A and TMT-B) to evaluate visual scanning, psychomotor speed and cognitive flexibility; and Stroop/Flanker tasks to investigate attentional control and inhibitory function. Visuospatial working memory and orientation were assessed by mental rotation and Corsi block-tapping activities (20, 21).

### Upper and lower-limbs neuromuscular performance

Neuromuscular performance was measured on days three and four, with lower-limb assessments performed on day three. Participants completed countermovement jump (CMJ) and isometric mid-thigh pull (IMTP) evaluations on dual K-Deltas force platforms (Kinvent Physio, Montpellier, France) with a sampling rate of 1,000 Hz. All measurement devices were calibrated before each testing session according to the manufacturers’ recommendations. Before neuromuscular testing, participants completed a standardized 10 min warm-up including light jogging, dynamic mobility exercises, progressive submaximal jumps and practice contractions specific to the tests. In CMJ trials, athletes performed maximal jumps with their hands on their hips, from which peak forces dominant, non-dominant and total were extracted from force–time curves. After a standard 60 s rest, IMTP trials consisted of a 5 s maximal pull on a stationary bar, with peak unilateral and bilateral forces recorded. The dynamic strength index (DSI) was the principal outcome, computed as the ratio of peak force during the CMJ to peak force during the IMTP (22, 23).

Unilateral strength asymmetry for both the CMJ and IMTP was assessed by calculating the percentage difference between dominant and non-dominant limbs using the formula [(D—ND)/D × 100]. Limb dominance was determined according to the greater peak force output during testing, consistent with the performance-based definition (22). Accordingly, the dominant limb was defined as the limb producing the greater peak force, whereas the non-dominant limb was the contralateral limb producing the lower peak force, irrespective of self-reported limb preference. Participants rested 60–90 s between repeated trials of the same assessment and at least 3 min between different neuromuscular assessments. Three maximal trials were performed for each neuromuscular assessment, with the highest peak force or jump height retained for analysis.

On the fourth day, eccentric hamstring strength was evaluated through the Nordic Hamstring Exercise (NHE). The force production from each lower-limb was measured using two K-Push devices (Kinvent Physio, Montpellier, France), which sampled at 250 Hz and were fitted with dual load cells. Participants engaged in a standardized 5 min dynamic warm-up focusing on trunk and lower-limb muscles prior to adopting a kneeling posture on a specialized NHE apparatus. Testing proceeded on a comfortable platform with arms crossed over the chest and a linear alignment from knee to head. Athletes were directed to lean forward gradually and carefully, reducing hip flexion while maintaining resistance against the descent until eccentric control was compromised.

The ankles were firmly secured above the lateral malleoli with braces linked to integrated load cells, recording eccentric-phase forces. Absolute maximum bilateral eccentric force (N) and inter-limb asymmetry were obtained from force-time data (24, 25). Bilateral maximal isometric grip strength was evaluated utilizing a hand-held dynamometer (K-Grip, Kinvent) (26, 27). Participants executed three maximal voluntary contractions each hand under controlled conditions with verbal encouragement, from which dominant, non-dominant and total peak forces were derived. Unilateral asymmetry was determined as the percentage difference between dominant and non-dominant limbs (22).

#### Statistical analysis

As this investigation used an exploratory Gaussian graphical model (GGM), there is presently no recognized *a priori* sample size technique for EBICglasso network estimation. Before initiating participant recruitment, a G*Power analysis (version 3.1.9.2; Heinrich Heine University, Düsseldorf, Germany) was carried out to determine the required minimum sample size for the overarching cross-sectional research based on a traditional fixed-effects ANOVA framework (*α* = 0.05, powe*r* = 0.80). This calculation was not intended as a justification for the suitability of the network model and is thus not taken as confirmation that the sample size is adequate for network estimation (28). According to current methodological recommendations (28, 29), the accuracy of network models is influenced by multiple factors, including network sparsity, boundary strength, variable distributions and regularization, instead of being determined by a fixed participant-to-variable ratio. Thus, the estimated network should be considered experimental. Descriptive data are presented as mea*n* ± standard deviation for anthropometric, maturational, cognitive and neuromuscular factors.

Before network estimation, data were screened for missing values, outliers, and distributional characteristics. Variables with potentially non-normal distributions (e.g., error counts, accuracy measures, and asymmetry indices) were examined using descriptive statistics and graphical diagnostics. Network-based performance analysis was conducted on the whole sample (*N* = 88) to explore conditional connections between maturational, anthropometric, neuromuscular and cognitive factors. To maintain statistical power, sport discipline was not included as a covariate because the available sample size was insufficient to support adjusted network estimation while maintaining reliable parameter estimation. The network was estimated using the Extended Bayesian Information Criterion graphical least absolute shrinkage and selection operator (EBICglasso; *γ* = 0.25) in which edges indicate regularized partial correlation coefficients adjusting for all other factors. The cor_auto function was used to estimate correlations and to automatically pick the proper correlation coefficient based on variable type.

Network estimation and visualization were performed using the qgraph package in R (version 4.3.3). Expected Influence was selected as the primary centrality measure because it accounts for both the magnitude and direction of edge weights, whereas Strength centrality was examined as a complementary measure owing to its established stability and interpretability in regularized Gaussian graphical models. Betweenness and Closeness were calculated for descriptive purposes only and were not considered primary inferential measures because these indices are generally less stable in regularized cross-sectional networks, particularly when sample sizes are modest. Network accuracy was evaluated using 2,000 nonparametric bootstrap resamples to estimate 95% confidence intervals for edge weights and centrality stability was assessed using a case-dropping bootstrap procedure across retained sample proportions of 90%, 70%, 50% and 40%. Stability was quantified using the correlation stability (CS) coefficient as recommended by Epskamp et al. 2018 (30).

## Results

**Figure 2:**
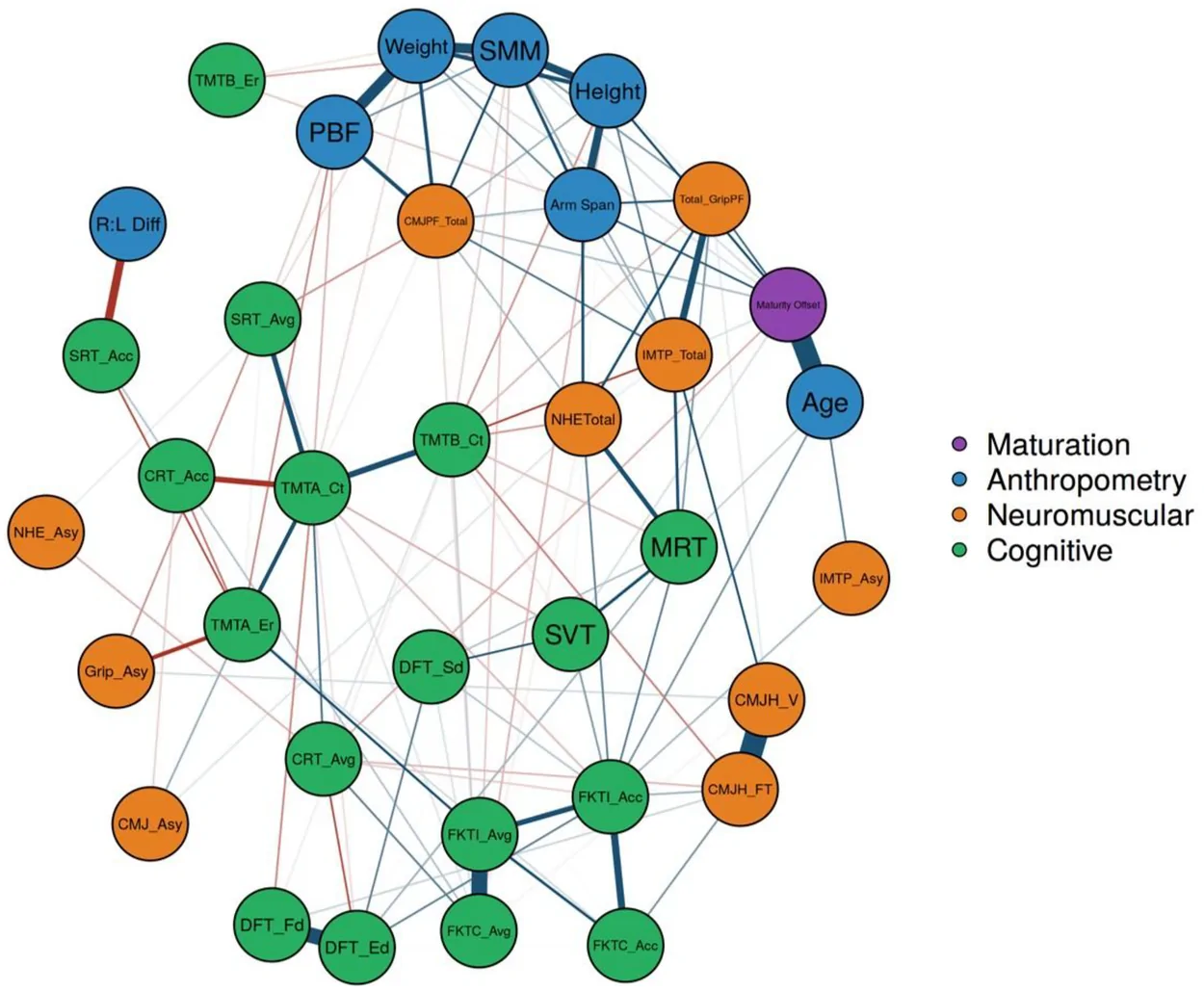
Estimated partial correlation network of maturational, anthropometric, neuromuscular and cognitive variables in youth combat athletes. Chronological age, chronological age; Maturity offset, years from peak height velocity; RF–LF, right-to-left 2D:4D difference; SRT_Avg, simple reaction time (average); SRT_Acc, simple reaction time accuracy; CRT_Avg, choice reaction time (average); CRT_Acc, choice reaction time accuracy; TMTA_Ct, Trail Making Test A completion time; TMTA_Er, Trail Making Test A errors; TMTB_Ct, Trail Making Test B completion time; TMTB_Er, Trail Making Test B errors; FKTC_Avg, Five-Key Task congruent reaction time; FKTC_Acc, Five-Key Task congruent accuracy; FKTI_Avg, Five-Key Task incongruent reaction time; FKTI_Acc, Five-Key Task incongruent accuracy; DFT_Fd, Design Fluency Test filled dots; DFT_Ed, Design Fluency Test empty dots; DFT_Sd, Design Fluency Test switching dots; DFT_Tc, Design Fluency Test total correct designs; MRT, Mental Rotation Test; SVT, Spatial Visualization Test; CMJ, countermovement jump; IMTP, isometric mid-thigh pull; NHE, Nordic hamstring exercise; PF, peak force; D, dominant limb; ND, non-dominant limb; DSI, dynamic strength index.

**Figure 3:**
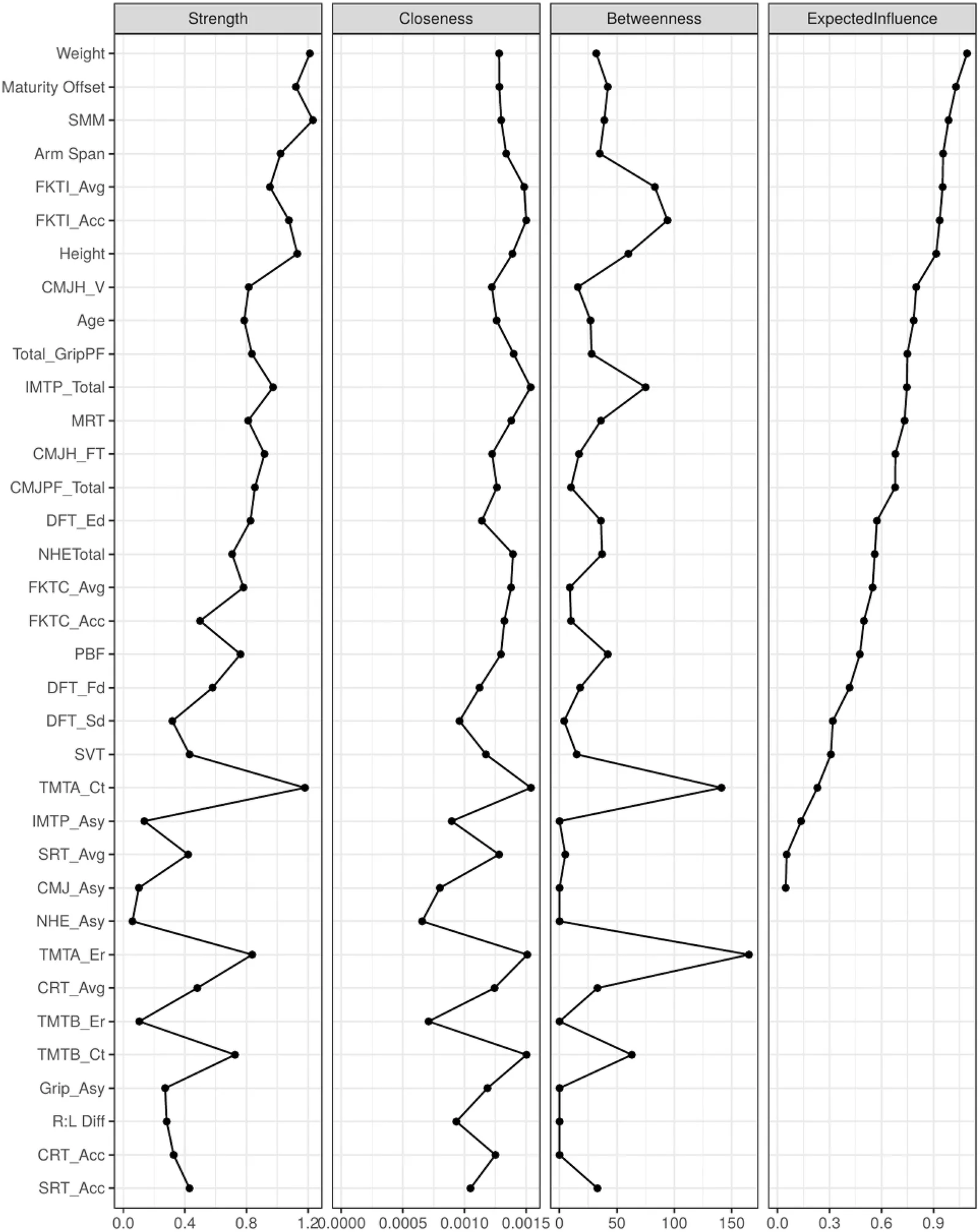
Standardized (z-score) centrality indices (strength, closeness, betweenness and expected influence) for all nodes in the estimated performance network.

The qgraph package's EBICglasso algorithm was used to estimate a Gaussian graphical model. The cor_auto function, which automatically chose the proper correlation coefficient based on the kind of variable, was used to calculate correlations (Figure 2). Performance Network Analysis revealed a connected network of 35 variables across the domains of maturation, anthropometry, neuromuscular performance and cognitive function (Figure 3). The EBICglasso model (*γ* = 0.25) estimated 129 out of 595 possible edges, corresponding to a network density of 21.7%. The network showed strong within-domain connectivity and several cross-domain conditional associations, suggesting important interactions between maturational, anthropometric, neuromuscular and cognitive characteristics.

Node centrality analysis revealed that anthropometric, maturational and selected cognitive variables occupied the most influential positions in the performance network (Table 2 and Figure 3). Strength centrality indicated that skeletal muscle mass (1.232), body mass (1.212), Trail Making Test A completion time (1.181), body height (1.131) and maturity offset (1.122) were among the nodes with the largest centrality estimates. Expected Influence showed a broadly similar pattern, with body mass (1.094), maturity offset (1.029), skeletal muscle mass (0.987), arm span (0.956) and Five-Key Task incongruent accuracy (0.936) showing the largest positive influence. Betweenness centrality identified Trail Making Test A errors (165) and completion time (141) as the principal highly connected nodes, followed by Five-Key Task incongruent accuracy (94), Five-Key Task incongruent reaction time (83) and total isometric mid-thigh pull peak force (75).

**Table 2:** Raw centrality measures of the fifteen most influential nodes in the estimated network, ranked according to strength centrality. .

Variables	Domain	Strength	Expected Infl.	Betweenness
Skeletal muscle mass (kg)	Anthropometric	1.232	0.987	39
Body mass (kg)	Anthropometric	1.212	1.094	32
TMTA_Ct (s)	Cognitive Function	1.181	0.230	141
Body height (cm)	Anthropometric	1.131	0.916	60
Maturity offset (yrs)	Maturation	1.122	1.029	42
FKTI_Acc (%)	Cognitive Function	1.077	0.936	94
Arm span (cm)	Anthropometric	1.023	0.956	35
IMTP_Total_PF (N)	Neuromuscular Performance	0.974	0.746	75
FKTI_Avg (%)	Cognitive Function	0.953	0.953	83
Height jump FT (cm)	Neuromuscular Performance	0.918	0.680	17
CMJ_Total_PF (N)	Neuromuscular Performance	0.855	0.678	10
TMTA_Er (n)	Cognitive Function	0.838	−0.039	165
Total grip strength_PF (N)	Neuromuscular Performance	0.835	0.749	28
DFT_Ed (n)	Cognitive Function	0.826	0.574	36
Height jump V (cm)	Neuromuscular Performance	0.815	0.800	16

Standardized (z-score) centrality values are presented separately in Figure 3 for visualization. TMT, Trail Making Test; Tc, total correct designs; FKT, Flanker Task; I, incongruent; IMTP, isometric mid-thigh pull; PF, peak force; Avg, average; Height jump FT, Jump height calculated using flight time; Height jump V, Jump height calculated via velocity-time integration; CMJ, countermovement jump; Er, error; PF, peak force; DFT, Design Fluency Test; Ed, empty dots.

**Figure 4:**
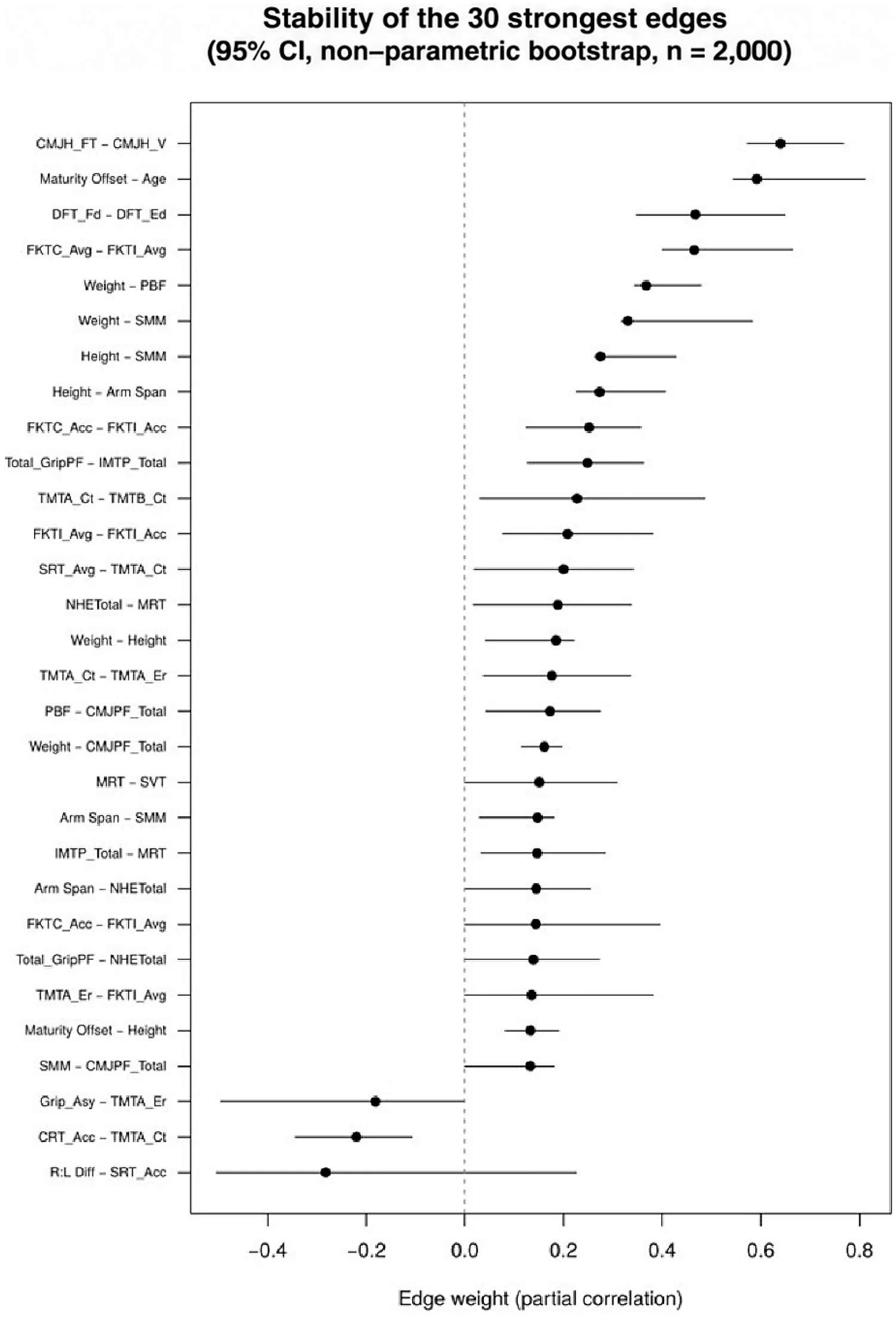
Bootstrap 95% confidence intervals for the 30 strongest partial correlation edges in the estimated network.

Bootstrap analysis identified the strongest partial correlations between countermovement jump height derived from flight time and take-off velocity (weight = 0.640; 95% CI = 0.572–0.768) and between maturity offset and chronological age (weight = 0.592; 95% CI = 0.543–0.811) (Table 3 and Figure 4). The Design Fluency Test filled and empty dots (0.468), Five-Key Task congruent and incongruent reaction times (0.465), body mass and percent body fat (0.368), body mass and skeletal muscle mass (0.330), body height and skeletal muscle mass (0.275) and body height and arm span (0.273) were among the other substantial positive associations.

**Table 3:** Selected partial correlation edges and bootstrap confidence intervals with bootstrap 95% confidence intervals.

Edge	Weight	95% CI
Height jump FT (cm)– Height jump V (cm)	0.640	0.572, 0.768
Maturity Offset (yrs)—Chronological age (yrs)	0.592	0.543, 0.811
DFT_Fd (n)—DFT_Ed (n)	0.468	0.347, 0.649
FKTC_Avg (s)—FKTI_Avg (s)	0.465	0.401, 0.665
Body mass (kg)—Percent body fat (%)	0.368	0.343, 0.479
Body mass (kg)—Skeletal muscle mass (kg)	0.330	0.317, 0.583
Difference (RF–LF) (mm)—SRT_Acc (%)	−0.282	−0.504, 0.226
Body height (cm)—Skeletal muscle mass (kg)	0.275	0.263, 0.429
Body height (cm)—Arm Span (cm)	0.273	0.226, 0.406
FKTC_Acc (%)—FKTI_Acc (%)	0.252	0.125, 0.357
Total_Grip_PF (N)—IMTP_Total_PF (N)	0.248	0.127, 0.363
TMTA_Ct (s)—TMTB_Ct (s)	0.227	0.031, 0.487
CRT_Acc (%)—TMTA_Ct (s)	−0.220	−0.345, −0.106
FKTI_Avg (s)—FKTI_Acc (%)	0.209	0.076, 0.381
SRT_Avg (s)—TMTA_Ct (s)	0.200	0.019, 0.341

Height jump FT, Jump height calculated using flight time; Height jump V, Jump height calculated via velocity-time integration; DFT, Design Fluency Test; Fd, filled dots; Ed, empty dots;; FKT, Flanker Task; C, congruent; I, incongruent; Avg, average; RF–LF, difference between right and left 2D:4D; SRT, simple reaction time; Acc, accuracy (% correct responses); PF, peak force; IMTP, isometric mid-thigh pull; SRT, simple reaction time; CRT, choice reaction time; TMT, Trail Making Test; Tc, total correct designs.

**Figure 5:**
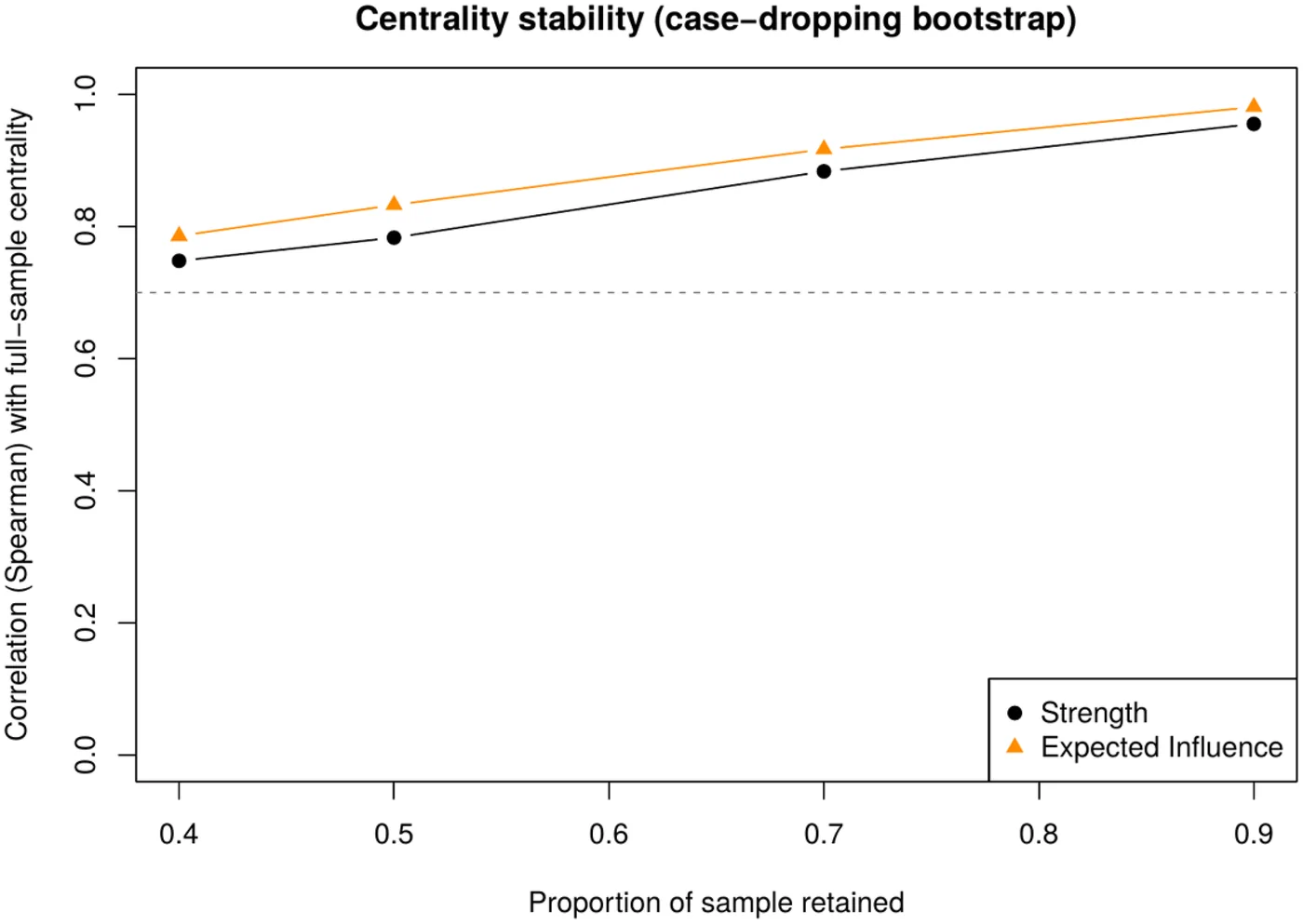
Case-dropping bootstrap analysis of centrality stability for strength and expected influence.

Several cross-domain conditional associations were also identified, including total grip strength and total isometric mid-thigh pull peak force (weight = 0.248; 95% CI = 0.127–0.363), Trail Making Test A and B completion times (weight = 0.227; 95% CI = 0.031–0.487) and Trail Making Test A completion time and simple reaction time (weight = 0.200; 95% CI = 0.019–0.341) were found to be cross-domain associations. Trail Making Test A completion time and choice reaction time accuracy were shown to be negatively correlated (weight = −0.220; 95% CI = −0.345 to −0.106). The connection between the right-to-left 2D:4D difference and simple response time accuracy (weight = −0.282; 95% CI = −0.504 to 0.226) was characterized by a wide confidence interval spanning zero and was therefore considered an imprecisely estimated edge rather than a robust network feature. Case-dropping bootstrap analysis demonstrated strong agreement between full-sample and subsample centrality estimates across all retained sample proportions (Figure 5). Correlation values for Expected Influence ranged from 0.786 to 0.981, whereas those for Strength ranged from 0.748 (40% retained sample) to 0.955 (90% retained sample). The Strength centrality index demonstrated acceptable stability (CS = 0.60) and the case-dropping bootstrap results indicated comparable stability for Expected Influence. The complete bootstrap stability results are presented in Figure 5.

## Discussion

The purpose of this study was to characterize the multidimensional relationships among anthropometric, maturational, neuromuscular and cognitive characteristics in trained youth combat athletes using network analysis. Contrary to our hypothesis, the executive cognitive and neuromuscular variables did not exhibit the greatest network centrality. Instead, the most highly connected nodes of the network were anthropometric and maturational variables, particularly skeletal muscle mass, body mass, body height and maturity offset, likely reflecting the overarching effects of adolescent growth and biological maturation on body size, muscle development and neuromuscular performance.

Cognitive variables such as Trail Making Test A completion time and Five-Key Task incongruent accuracy remained substantially related, with moderately high Strength, Expected Influence and Betweenness values. However, because of the fact that bridge-centrality measures were not derived from pre-defined communities, these variables should be interpreted as strongly linked and not formally highly connected. Overall, our results suggest the multidimensional performance network in this cohort was mostly structured by developmental and morphological features.

The results suggest that performance development in young combat athletes is organized as an integrated multidimensional system and not as independent physical and cognitive components. Moreover, the network reproduced recognized developmental relationships between growth, body composition and maturation while demonstrating cross-domain relationships with executive function, supporting the face validity of the network and highlighting that executive cognitive variables were conditionally associated with multiple domains within the estimated performance network.

The predominance of anthropometric and maturational variables was expected biologically and demonstrates that the network has learned well established processes of development in adolescence. The strong correlations among body height, body mass, skeletal muscle mass and maturity offset are reflective of the synchronized growth that takes place during biological maturation and peak height velocity (13, 18). These developmental changes underpin the improvements in strength and physical performance and are recognized as important determinants of long-term athlete development (1, 6).

This finding is consistent with the positive relationships between body mass, skeletal muscle mass, body height and arm span found in young combat athletes (5, 9), which are consistent with the morphological changes observed. However, from a network perspective, the high centrality of these variables indicates their broad conditional associations within the performance system rather than a direct causal influence on athletic performance (14). Collectively, these results emphasize the need to consider biological maturation when assessing neuromuscular performance and interpreting multidimensional performance profiles in young combat athletes (13, 15).

Besides the expected developmental structure, the most striking finding was the central bridging role of executive cognitive variables in the performance network. Trail Making Test A completion time demonstrated high Strength and relatively high Betweenness values, whereas Five-Key Task incongruent accuracy showed high Expected Influence. Because Strength demonstrated acceptable stability (CS = 0.60), interpretation primarily focused on Strength and Expected Influence. Betweenness values are presented only as descriptive network characteristics and should not be interpreted as evidence of highly connected centrality (14). The results suggest that processing speed, attentional control and inhibitory function are conditionally related to various performance domains, but the cross-sectional design does not allow for causal conclusions.

This interpretation aligns with evidence that successful performance in combat sports is based on the integration of executive function with neuromuscular capacity to support rapid perception, decision-making and tactical responses under competitive demands (3, 4, 7, 12). The observed network structure suggests that executive cognitive function and neuromuscular performance are conditionally interconnected within this sample. These findings generate hypotheses regarding the potential value of integrated cognitive and neuromuscular training approaches; however, longitudinal and intervention studies are required before specific training recommendations can be made (4, 8, 21). However, network centrality is a measure of structural connectivity, not causal importance and longitudinal studies are necessary to determine whether these cognitive variables impact, or simply co-develop with, physical performance (14).

Edge-level analysis revealed several strong conditional associations that were physiologically plausible and consistent with previous evidence. Strong associations were found between maturity offset, anthropometric features and neuromuscular performance, which suggests the co-occurrence of biological maturation, body size, muscular strength and lower-limb power during adolescence (5, 9, 26, 27). Moreover, the association between the time to complete the Trail Making Test A and the accuracy of choice reaction time supports the strong relationship between processing speed and executive control reported in athletes with higher perceptual-cognitive expertise (3, 7).

Not all edge-weight estimates were characterized by the same level of precision. Consistent with current recommendations for regularized network models, bootstrap confidence intervals were interpreted as indicators of estimation precision rather than as hypothesis tests (14). Consequently, edges associated with relatively wide confidence intervals, including the association between the right-to-left 2D:4D difference and simple reaction time accuracy, should be regarded as uncertain and interpreted cautiously until replicated in independent samples. Accordingly, interpretation focused primarily on stable characteristics of the estimated network, including the overall network organization, cross-domain conditional associations and highly connected nodes demonstrating acceptable stability, rather than on isolated edge estimates (14, 28).

Several methodological considerations should be taken into account when interpreting the present findings. First, the cross-sectional design precludes definitive causal inferences regarding the conditional relationships among variables or the structural organization of the estimated performance network. Although the network demonstrated acceptable Strength centrality stability (CS = 0.60), it was estimated from 35 variables in a relatively modest sample (*N* = 88). While EBICglasso regularization reduces the risk of overfitting by shrinking weaker associations toward zero, it cannot eliminate uncertainty in edge estimation or guarantee recovery of the underlying population network. Consequently, highly central nodes should be interpreted as variables occupying structurally important positions within the estimated network rather than as causal determinants of athletic performance or priority targets for intervention.

Furthermore, several nodes represented mathematically related or derived measures, including body composition indices, unilateral and total force outputs, alternative calculations of countermovement jump height and derived Trail Making Test variables. These measures were intentionally retained because the aim of the present exploratory study was to characterize the multidimensional athlete profiling framework as implemented in applied practice rather than to construct a parsimonious network of statistically independent variables. Nevertheless, some of the strongest within-domain associations—particularly between variables derived from the same measurement or computational procedure—are likely to reflect mathematical coupling or shared measurement characteristics in addition to underlying biological relationships.

For this reason, greater interpretive emphasis should be placed on the overall network structure and the cross-domain conditional associations linking anthropometric, maturational, neuromuscular and cognitive characteristics than on isolated within-domain edges. Future studies should evaluate reduced-variable network models and formally compare their topology with the present network to determine the extent to which mathematically related variables influence edge weights, centrality estimates and the robustness of the identified performance structure. Lastly, biological maturation was estimated using a maturity-offset equation rather than direct assessment of maturity offset status. As this method is subject to individual prediction error, the maturational findings should be interpreted with caution.

## Conclusion

This study demonstrates that Performance Network Analysis is a systems-based framework to describe the multidimensional organization of multidimensional performance characteristics in trained youth combat athletes. Anthropometric and maturational characteristics emerged as structural of the performance network, with executive cognitive function serving as an important node connecting cognitive, neuromuscular and physical domains. These results suggest that performance development during adolescence is a consequence of the interaction of biological maturation, physical capacities and executive function and not a function of individual performance attributes. From an applied and practical perspective, the present findings illustrate how network analysis can complement multidimensional athlete profiling by describing the conditional relationships among maturational, anthropometric, neuromuscular and cognitive characteristics. However, because the present study was cross-sectional, these findings should not be interpreted as identifying causal mechanisms, individualized training targets, or prospective indicators of talent development. Instead, the estimated network provides a systems-based framework for generating hypotheses to be examined in future longitudinal and intervention studies.

## Data Availability

The raw data supporting the conclusions of this article will be made available by the authors, without undue reservation.
